# Femtogram Level Sensitivity achieved by Surface Engineered Silica Nanoparticles in the Early Detection of HIV Infection

**DOI:** 10.1038/s41598-017-07299-1

**Published:** 2017-08-02

**Authors:** L. A. Avinash Chunduri, Aditya Kurdekar, Mohan Kumar Haleyurgirisetty, Eswarappa Pradeep Bulagonda, Venkataramaniah Kamisetti, Indira K. Hewlett

**Affiliations:** 1 Laboratories for Nanoscience and Nanotechnology Research, Sri Sathya Sai Institute of Higher Learning, Prasanthi Nilayam, 515134 Andhra Pradesh India; 2 Department of Biosciences, Sri Sathya Sai Institute of Higher Learning, Prasanthi Nilayam, 515134 Andhra Pradesh India; 3 Laboratory of Molecular Virology, Centre for Biologics Evaluation and Research (CBER), US Food and Drug Administration, Bldg. 72, White Oak Campus, 10903 New Hampshire Avenue, Silver Spring, MD 20993 USA

## Abstract

We have engineered streptavidin labelled Europium doped fluorescent silica nanoparticles which significantly increased sensitivity without compromising the specificity of the immunoassay. As a proof of concept, a time resolved fluorescence based sandwich immunoassay was developed to detect HIV-1 p24 antigen in clinical specimens. The detection range of the silica nanoparticle based immunoassay (SNIA) was found to be between 0.02 to 500 pg/mL in a linear dose dependent manner. SNIA offers 1000 fold enhancement over conventional colorimetric ELISA. Testing of plasma samples that were HIV negative showed no false positive results in the detection of HIV-1 p24 antigen. This highly sensitive p24 assay can help improve blood safety by reducing the antibody negative window period in blood donors in resource limited settings where nucleic acid testing is not practical or feasible. This technology can also be easily transferred to a lab-on-a-chip platform for use in resource limited settings and can also be easily adopted for the detection of other antigens.

## Introduction

World Health Organization estimates that more than 37 million people are living with HIV, of whom 2.1 million are new infections and 1.4 million died of AIDS in 2015, a number which is growing annually^1^. The only way to tackle this growing global concern is by immediate diagnosis of the disease in the infected individuals and spreading awareness to prevent further infection. To that effect, implementation of HIV testing has helped lower incidence and transmission rates during the past decades^2^. However, most HIV tests are lab-based, highly automated, sophisticated, and requires training. There is a need for platforms adaptable for point-of-care use, multiplexing and rapid modification for high sensitivity detection of emerging pathogens.

Nanotechnology could potentially provide a new generation of assays for rapid, highly sensitive and multiplexed detection of pathogens in a miniaturized format. While nucleic acid testing (NAT) is the most sensitive method to detect HIV in the early phase of infection, current assay formats are not always feasible or practical in most settings^3^. The detection of viral protein, HIV-1 p24 antigen, using immunoassay techniques is an alternate method for early detection^4^. Further, immunoassays are easier to perform and more adaptable to POC^5^.

Fluorescent nanoparticles are becoming increasingly important to improve sensitivity, specificity and multiplexing capacity over conventional fluorophores in the field of biosensors and bioimaging^6, 7^. High fluorescent emission, well known surface functionalization methods, low cytotoxicity and reliable scale up procedures are the attracting features for various *in vitro* and *in vivo* applications^8^. Various fluorescent nanoparticles such as lanthanide doped nanoparticles, metal-based dyes, dye impregnated nano and microparticles, luminescent nanoparticles, semiconductor quantum dots, and noble metal nanoclusters have been exploited for various biological applications^9^. Among these rare earth doped silica nanoparticles have attracted much attention due to their high flexibility for conjugating wide range of biomolecules, their biocompatibility and monodispersity^10^. These rare earth doped silica nanoparticles exhibit advantages of narrow emission spectrum, well separated excitation and emission spectra and long luminescence life time^11^. So, based on these feature we can use time resolved fluorescence (TRF), wherein the emission is measured as a function of time, can be used in to increase the detection sensitivity in biological samples due to its high signal to noise ratio^12^. To our knowledge there are no reports on the application of europium doped silica nanoparticles for fluorescence based immunoassays.

Diagnosis of HIV-1 infection, the cause of AIDS, relies on the detection of HIV-1 RNA, capsid antigen (p24), and anti HIV antibody^13^. High levels of p24 antigen are found during the early phase and also in the terminal stage of the AIDS^14^. It is also a useful marker for monitoring patient treatment and also for testing blood samples in the regions where HIV-1 RNA testing is not available. HIV-1 p24 antigen is usually detected by enzyme-linked immunosorbent assay (ELISA) whose detection sensitivity is 10–20 pg/mL^15^. The conventional HIV-1 p24 ELISA is an enzyme-based colorimetric assay that involves multiple steps of incubation. The past decade has seen significant improvement in the HIV-1 p24 antigen assays. This improvement can be attributed to implementation of immune complex disruption methods, using more efficient lysis buffers, and incorporation of tyramide-mediated boosting techniques in the assay^16^. Lower detection limits of 1 pg/mL have been achieved for p24 antigen using the boosted ELISA^17^. Another sensitive method, real-time immuno-Polymerase Chain Reaction (immuno-PCR), can detect 1000 HIV-1 RNA copies, or 40 attogram of HIV-1 p24 antigen, per reaction^18^. However, all these improvements of detection sensitivity increase the complexity of testing which makes their deployment in Resource Limited Settings (RLS)﻿ infeasible.

With the goal of improving public health through early and accurate disease diagnosis, there is an increasing need for sensitive, simple, affordable and rapid detection of infectious disease biomarkers to enable faster access to test results and improved patient outcomes. The development of rapid, portable and accurate Point-of-Care (POC) clinical diagnostics could lower transmission rates, especially in cases of recent HIV infection in resource limited s﻿ettings by providing timely access to intervention^19^.

Although currently available detection methods exceed an accuracy of more than 99% targeting HIV antibodies produced by the host, they are suboptimal because it takes three weeks to six months to detect the antibodies^20^. This depends on the individual’s immune system which may result in delay in treatment. Several reports indicate that targeting the virus itself and particularly the HIV-1 capsid protein, p24 antigen could be an excellent diagnostic marker as the detectable levels in the host are very high immediately after infection^21, 22^. p24 being highly specific for HIV infection offers a major advantage for early diagnosis of HIV infected patients. HIV-1 p24 antigens appear at an earlier stage of HIV infection than antibodies, which is due to an explosive replication of the virus following acute infection and is correlated with highly infectious viraemia. Early detection of HIV-1 p24 would be of great value in the early detection of HIV infection, blood screening, neonatal HIV infection, and the surveillance of therapeutic efficacy and disease progression. However, after acute HIV infection, specific antibodies occurring in the body combine with p24 antigens to form immune complexes, causing the free antigen concentration to become too low to be detectable, this period is called “window phase”. Although it cannot be detected during this period, there is already HIV existing in the body and can be passed on to others. Therefore, there is a need to develop a novel highly sensitive method to directly identify p24 antigens at window phase. Thus, the p24 antigen can be used as an excellent biomarker in the diagnosis of HIV to efficiently shorten the “window period”. In our present work, we have chosen p24 as the biomarker for the early and sensitive identification of HIV infection by using fluorescent silica nanoparticles.

We have engineered streptavidin labeled fluorescent silica nanoparticles, for the development of a Silica Nanoparticle Immuno Assay (SNIA) for early and sensitive detection of HIV infection. As a proof of concept SNIA was demonstrated to detect HIV-1 p24 antigen in clinical specimens with a detection range between 0.02 to 500 pg/mL in a linear dose dependent manner. SNIA demonstrated a 1000 fold enhancement over conventional colorometric ELISA for detection of HIV p24 antigen. We have successfully demonstrated the detection of femto gram level concentration of HIV-1 p24 antigen using the SNIA. The specificity of the immunoassay was 100% when tested with plasma samples negative for HIV-1 p24 antigen and those infected with other viruses such as HBV, HCV and Dengue. Our findings confirm that fluorescent nanoparticle based immunoassays could dramatically increase detection sensitivity and have applications for improved diagnosis and point-of-care use. SNIA could be developed into a universal labelling technology by replacing capture and detection antibodies for detection of antigens of various pathogens in the future. There is no need for specific training of technicians to perform SNIA as it is similar to traditional ELISA which is widely used in various laboratories and clinics. Upon further optimization and simplification, this could be developed into a rapid and ultrasensitive testi﻿ng platform for clinical disgnostics and laboratory research.

## Methods

### Synthesis of europium doped silica nanoparticles by sol-gel Method

In a typical synthesis, silica nanoparticles doped with europium (SiO_2_:Eu NPs) were prepared using the modified Stober method^23^. 4 mL of Tetraethoxysilane (TEOS) (Sigma- Aldrich) was added to a mixture of 3.3 mL Ammonium Hydroxide (Sigma-Aldrich), 0.52 g of europium chloride (EuCl_3_) (Sigma Aldrich) and 47 mL of ethanol (Sigma Aldrich) with stirring and the reaction was continued for 24 hours. Nanoparticles were then washed with ethanol several times for purification and subsequently separated by centrifugation. The nanoparticle precipitate was dried under vacuum at 80 °C for 6 hours and annealed at 700 °C for 2 hours in air to favor Eu activation and to remove water and organic residuals from the material. Double distilled water was used for performing all the synthesis.

### Preparation of carboxylic acid functionalised SiO_2_:Eu NPs

SiO_2_:Eu were grafted with carboxyl groups in 2 steps.

In the first step amino-functionalized silica nanoparticles (SiO_2_-NH_2_) were prepared by a silanization process with APTES. In the next step, a ring opening linker elongation reaction of the amine functions with succinic anhydride to finally functionalise the silica surface with carboxyl groups^24^.

Non-aqueous system was chosen to prevent the hydrolysis and condensation of APTES. In a typical procedure, to functionalise with amine groups 83.75 μL of APTES was added drop by drop to the 300 mg of the SiO_2_:Eu nanoparticles dispersed in 15 mL of ethanol. The reaction was continued over night with vigorous stirring following which the nanoparticles were washed with ethanol and water and dried under vacuum for 8 hours to obtain SiO_2_:Eu-NH_2_ NPs.

In the next step 100 mg of SiO_2_:Eu-NH_2_ NPs were dispersed in 20 mL of DMF and added drop-wise to a mixture containing 5 mL of 50 mg succinic anhydride (Sigma-Aldrich) in DMF with vigorous stirring. Stirring was continued for 24 hours and the resulting silica nanoparticles with carboxylic functional groups were washed multiple times with ethanol and dried under vacuum at 60 °C for 8 hours. Double distilled water was used in all preparations.

### Bioconjugation of streptavidin to carboxylic functionalised SiO_2_:Eu nanoparticles

In our present study 1-Ethyl-3-(3-dimethylaminopropyl) carbodiimide (EDC)/Sulpho N-hydroxysuccinimide(Sulpho NHS) method was adopted to conjugate carboxyl functionalised silica nanoparticles covalently with primary amines of streptavidin as reported^25^. In a typical synthesis, 20 mg of nanoparticles were dispersed in 10 mM phosphate (pH = 7.0) buffer and washed in a NanoSep centrifugal ultrafiltration device with a molecular weight cutoff of 300 kDa (Pall Life Sciences, Ann Arbor, MI, USA). Nanoparticles were redispersed in phosphate buffer and the solution was sonicated with a tip sonicator for 30 secs. The carboxyl groups are activated with 10 mmol/L of EDC (Thermo Scientific, USA) and 10 mmol/L sulpho-NHS (Thermo Scientific, USA) in PBS buffer for 30 min. Activated particles were washed once with glycine buffer. 50 μL of 1 mg/mL Streptavidin (Scripps Lab, USA) in the carbonate buffer (pH = 9.0) solution was added. After 3 hours of incubation at room temperature the streptavidin conjugated nanoparticles were washed 5 times with glycine buffer. They were resuspended in DMSO and stored at 4 °C.

### SNIA for detection of HIV-1 p24

**Figure 1:**
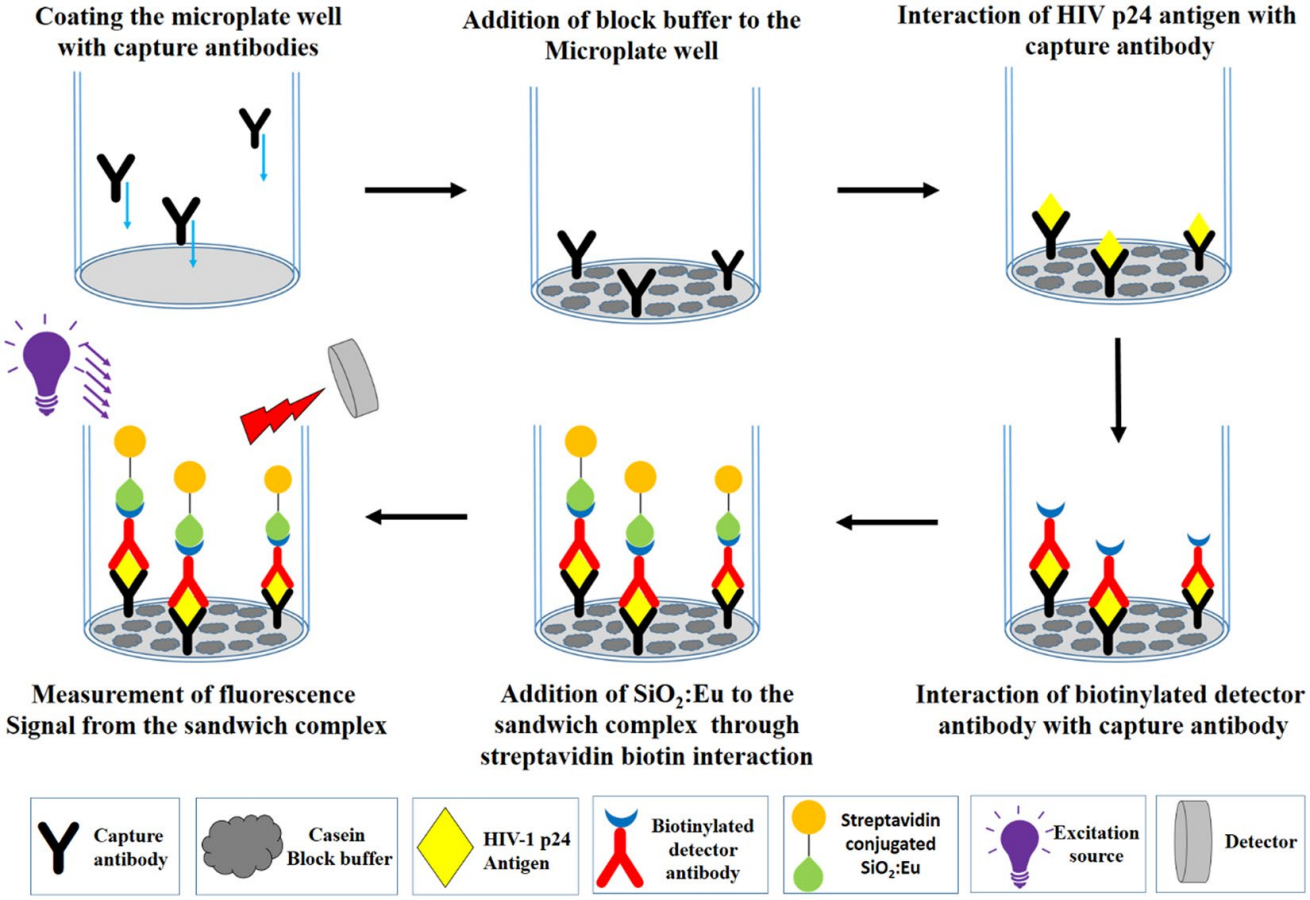
Schematic illustration of SNIA for detection of HIV-1 p24 antigen. HIV-1 p24 is bound to capture antibody which was coated on microtiter wells and then sandwiched with secondary biotinylated anti-p24 antibody. The immunocomplex is then coupled to Streptavidin conjugated silica nanoparticles, which recognizes and binds to the above biotinylated antigen–antibody complex. After extensive washing, the fluorescence signal released from the complex is then recorded and quantified with Spectramax M5 multiplate reader.

Our silica nanoparticle based immunoassay adopted the sandwich immunoassay format to capture the target (HIV-1 p24 antigen) which was described in an earlier communication for Europium nanoparticles^26^. 55 μL of Anti HIV-1 p24 Capture antibody (Prospec, USA) (2 μg/mL) diluted with carbonate-bicarbonate buffer (100 mM, pH 9.6) was coated on Nunc maxisorp fluorescence microplates and incubated for 24 hours at 4 °C. They were washed 5 times with wash buffer after which 300 μL/well Casein Blocking Buffer (Thermo Scientific, USA) was added to each well and discarded. Next, 250 μL/well of blocking buffer was incubated for 30 minutes at 37 °C to ensure the blocking of nonspecific adsorption sites on the microplate well. At this step washing was avoided. In the next step different concentrations (100 μL/well) of purified HIV-1 p24 antigen (Virogen, USA) diluted in assay buffer or diluted plasma samples were added to each well and incubated at 37 °C with shaking for 1 hour. The plasma samples were spiked into blocking buffer containing 55 μ﻿L of 10% Triton-X-100 per mL. After washing all the wells for 5 times with washing buffer, 100 μL/well of biotinylated HIV-1 p24 detector antibody (Perkin-Elmer, USA) was added and incubated for 30 minutes at 37 °C. To this antibody-antigen-antibody complex, 100 μL of Streptavidin conjugated europium doped silica nanaprticles diluted in DMSO were added. The mixture was incubated again for 30 minutes at 37 °C with shaking followed by final washing with PBST buffer for 5 times to avoid nonspecific interactions and reduce background noise. Finally, fluorescence was measured in a time resolved mode (with exitation at 268 nm and emission at 615 nm, decay time 400 μs, measurement window 400 μs). The complete schematic is presented in Fig. 1. All the experiments were performed in triplicates. The fluorescence cutoff value was derived using the sum of the means of the signal intensity of six negative controls plus 2 times their standard deviation (SD). Samples with signal-to-cutoff (S/CO) ratios equal to or greater than 14.00 were considered positive for HIV-1 p24.

## Results

### Characterization of annealed and functionalised SiO_2_ nanoparticles

#### FESEM analysis

**Figure 2:**
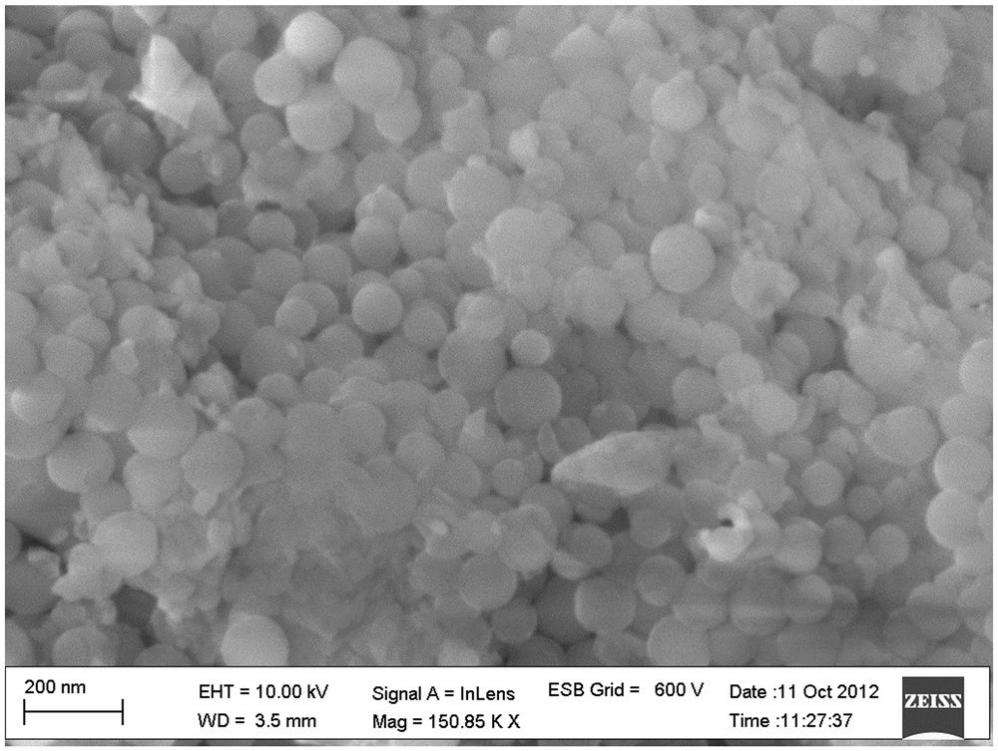
FESEM image of silica nanoparticles where the spherical morphology is clearly evident. The average size of the particles is around 60 nm.

Morphology, size and extent of aggregation were investigated by Field Emission Scanning Electron Microscope (FESEM), Zeiss Gemini Ultra 55, operated at 5 kV. FESEM analysis of annealed SiO_2_:Eu nanoparticles confirmed the size of these nanoparticles to be between 50–70 nm (Fig. 2). These nanoparticles were found to be spherical in shape even after thermal treatment. The problem of precipitation of rare earths as hydroxides in a basic pH environment was overcome in the present synthesis of europium doped silica nanoparticles and no sponge like precipitates were observed confirming the optimum concentration of europium for the formation of spherical nanoparticles.

#### Fourier Transform Infrared (FTIR) analysis

**Figure 3:**
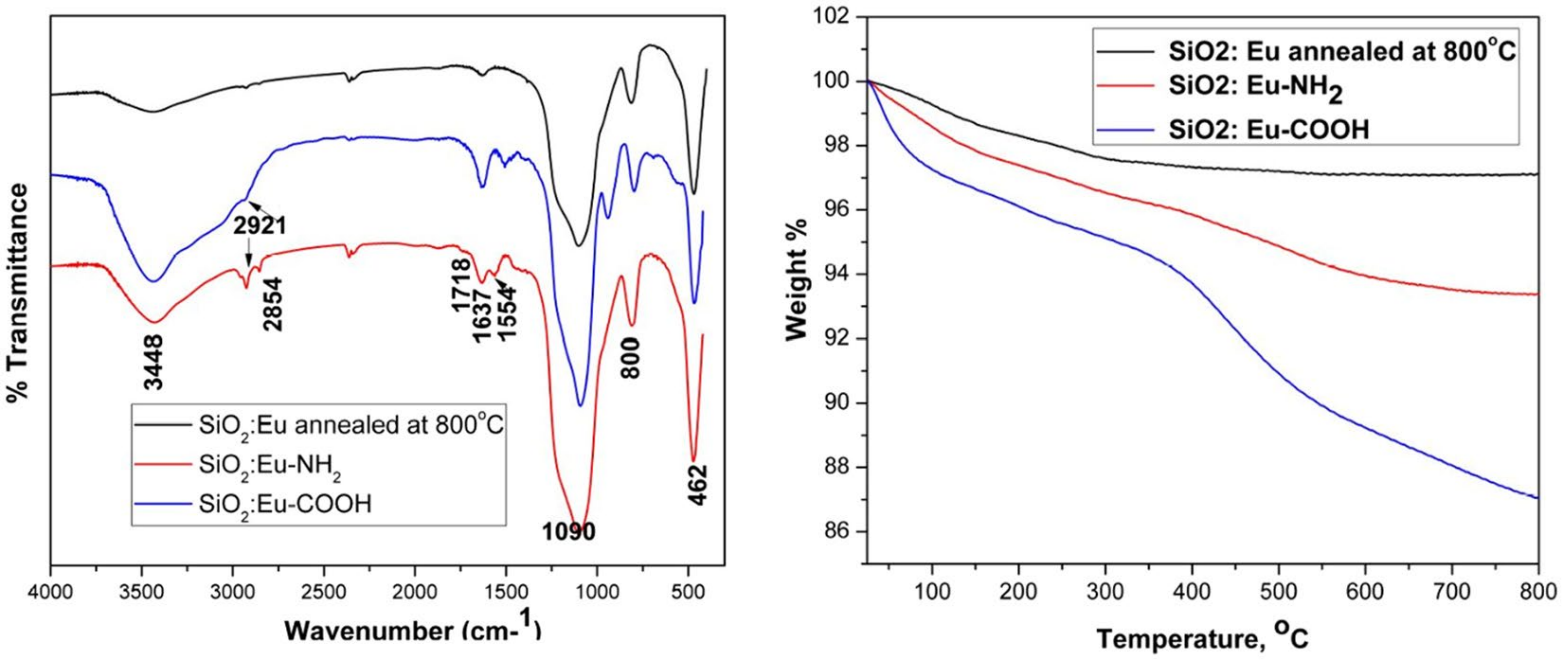
(**a**) FTIR spectra of annealed SiO_2_:Eu, SiO_2_:Eu-NH_2_ and SiO_2_:Eu-COOH functionalised nanoparticles (**b**) TGA curves of annealed SiO_2_:Eu, SiO_2_:Eu-NH_2_ and SiO_2_:Eu-COOH functionalised nanoparticles.

Surface composition of nanoparticles post functionalization was confirmed using Fourier Transform Infrared (FTIR) spectroscopy where the KBr pellet method was adopted. All FTIR spectra were collected using SHIMADZU IR Affinity-1, in the wavenumber range of 400–4000 cm^−1^ in transmission mode with an accumulation of 32 time scans and resolution of 2.0 cm^−1^. FTIR spectra of annealed europium doped SiO_2_, NH_2_ and COOH functionalised SiO_2_: Eu were recored to study the structure and functionalization (Fig. 3(a)). The bands at 1090 and 1220 cm^−1^ originating from asymmmetric and symmetric stretching vibrations of the Si-O-Si were observed in all the three samples. The band at 800 cm^−1^ indicates the presence of Si-OH stretching vibrations^27^. The bands observed at 2921 and 2851 cm^−1^ in amine functionalised samples resulting from assymetric stretching vibrations of the –CH_2_ groups confirmed the prescence of aminopropyl groups of APTES. The bands at 3450 and 1650 cm^−1^ were related to the stretching and bending vibrations of both the aliphatic amine groups present on the surface and silanol group^28^. The appearance of bands at 1718 and 1554 cm^−1^ observed in the carboxyl functionalised sample supports that -COOH groups were bonded to the surface through a reaction of -NH_2_ and succinic anhydride^29^.

#### Thermogravimetric Analysis

Thermogravimetric analysis (TGA) was performed to assess the extent of surface modification. TGA was carried out in air at a heating rate of 10 K min^−1^ till 800 °C using Mettler Toledo, TGA/DSC 1. Figure 3(b) shows the weight loss curves of annealed SiO_2_:Eu, SiO_2_:Eu-NH_2_, and SiO_2_:Eu-COOH respectively. The weight loss of all the samples was calculated and tabulated in Table 1. All the three samples exhibited two step weight losses. The first weight loss below 120 °C was due to the evaporation of physically adsorbed water and the second weight loss from 120 °C to 800 °C was attributed to the decomposition of organic matter. The weight loss for SiO_2_ sample was 2.91% and considered as reference for the calculation of surface functional groups of the other two samples. Decomposition of amino propyl groups led to higher weight loss (6.7%) in SiO_2_-NH_2_ sample compared to SiO_2_. The difference in weight loss of 3.79% is directly proportional to the degree of modification of the surface of nanoparticles.

**Table 1:** The weight loss percentages calculated from TGA for SiO_2_:Eu, SiO_2_:Eu-NH_2_ and SiO_2_:Eu-COOH functionalized nanoparticles.

Sample	Weight loss (Wt%)	Difference in wt loss (Wt%)
SiO_2_:Eu	2.91	—
SiO_2_:Eu-NH_2_	6.70	3.79
SiO_2_:Eu-COOH	13.10	6.40

Further, increased weight loss was observed in SiO_2_:COOH sample (13.1%) compared to SiO_2_-NH_2_ sample. This might be due to the increase in the organic content resulting from the reaction of SiO_2_:NH_2_ with succinic anhydride. The 6.4% difference in weight loss for SiO_2_:COOH can be attributed to the presence of well finctionalised -COOH groups on the surface. Our results thus confirm that the surface of silica nanoparticles were successfully modified with amine and carboxyl groups.

#### Optical Characterization

**Figure 4:**
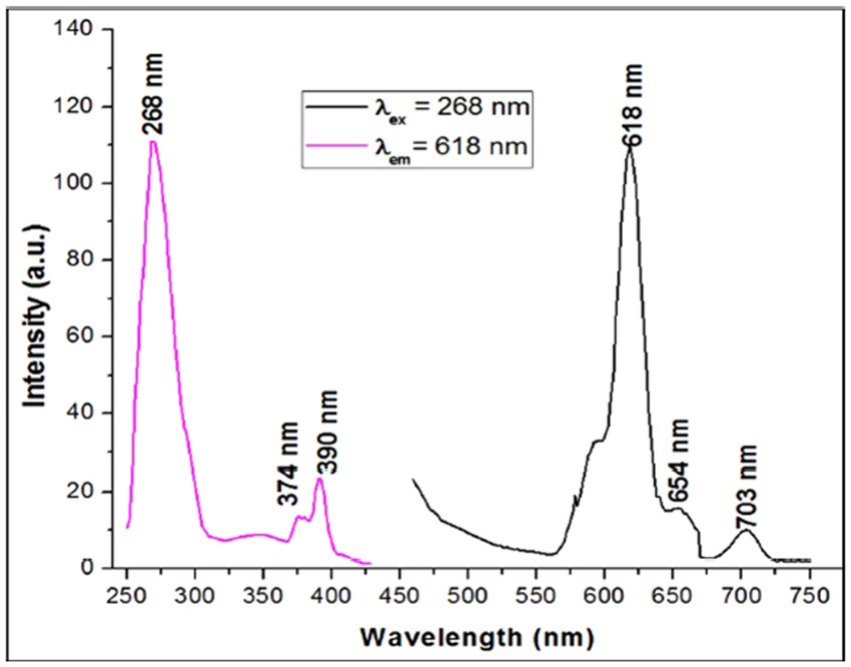
Excitation and Emission spectra of annealed SiO_2_:Eu nanoparticles. The peak at 268 nm is an absorption peak observed which when used as an excitation wavelength resulted in fluorescence emission at 618 nm.

Photoluminescence excitation and emission properties of the as synthesised Europium doped silica nanoparticles in the UV and visible regions (250–850 nm) were measured by Molecular Devices, SpectraMax M5 multimode fluorescence spectrophotometer. The same reader was also used to measure fluorescence signal for the detection of HIV-1 p24 antigen during immunoassay. Figure 4 displays the photoluminescence excitation and emission spectra of silica nanoparticles doped with Europium annealed at 700 °C. The emission spectrum was obtained by exciting the sample at 268 nm and the excitation spectrum was obtained by monitoring the 618 nm emission peak. Upon excitation, a strong narrow red emission was observed at 618 nm along with 3 more weak emission peaks located at 595, 657 and 705 nm. The 618 nm emission peak has been assigned to ^5^D_0_ → ^7^F_2_ transitions of Eu^3+^ ions and the remaining 3 peaks were assigned to the f-f transitions of the localized rare earth ions^30^. A broad emission band in the lower wavelength region from 400 nm to 500 nm was observed in addition to narrow emission peaks reported previously^31^. These broad peaks were common in organically prepared silica nanoparticles. The excitation spectrum reveals the presence of intense absorption bands in the UV region, at 268, 374 and 390 nm.

In this study, in addition to the well established methods for functionalization and bioconjugation of streptavidin to silica, the aspects of large Stokes shift, narrow emission from europium ions had motivated us to choose this particular material for HIV-1 p24 detection.

### Silica Nanoparticle based Immunoassay (SNIA) for HIV-1 p24 detection using time resolved fluorescence

HIV-1 p24 antigen is the target viral protein in this study because of it abundance in the early stages of HIV infection even when the antibody concentrations are still low. This, in turn, helps in early detection of the disease which can allow its better management and antiretroviral treatment. We adopted p24 antigen assay to study the SNIA performance. In order to test the sensitivity of the SNIA, we decided to test its limits in detecting p24 antigen. The immunoassay involved the formation of antibody-antigen-antibody sandwich complex where in the detection step involved interaction between the biotinylated detector antibody and streptavidin conjugated Silica Nanoparticles^32^. The detection process makes use of the strong non-covalent chemistry between biotin and streptavidin which immobilizes the fluorophore to the sandwich complex^15^. When illuminated by excitation energy, the fluorophore emits and the signal intensity is proportional to the amount of streptavidin conjugated silica nanoparticles. The amount of streptavidin conjugated silica is proportional to amount of p24 present. Thus, the signal intensity is indirectly proportional to the concentration of p24 present in the sample. The Fluorescence signal intensity was then measured using a SpectraMax M5 microplate reader. A calibration curve for the purified HIV-1 p24 antigen was plotted with the measured data.

### Calibration curve

**Figure 5:**
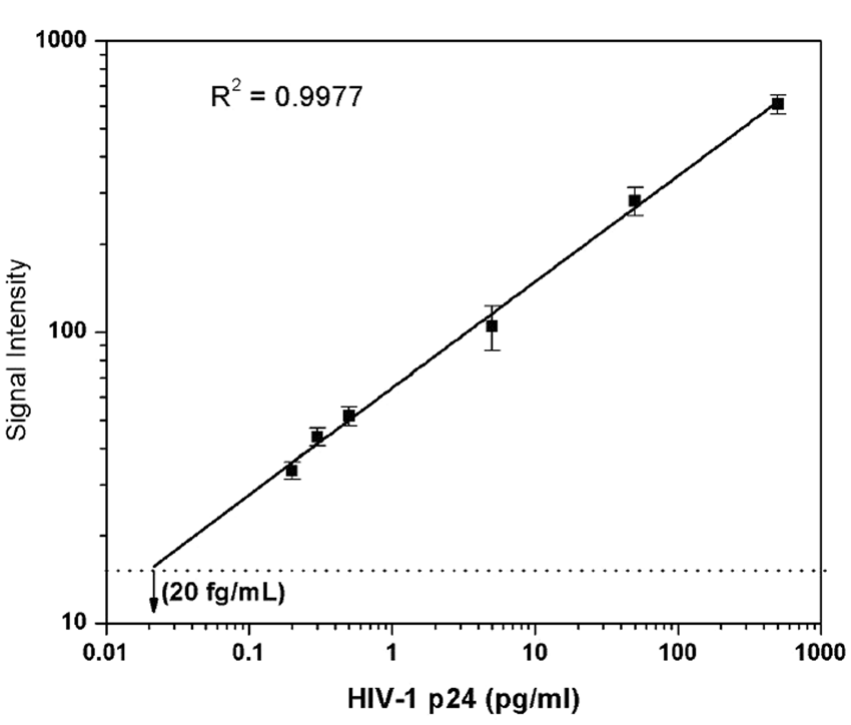
Calibration curve of silica nanoparticle based immunoassay (SNIA) for the detection of HIV-1 p24 antigen (n = 3). Purified HIV-1 p24 antigen (range, 0.02 pg/mL to 1000 pg/mL in serial dilution in PBS) served as target. The normalized relative signal intensities are represented as the ratios of samples over the cutoff value of the negative control. The dotted line (---) represents the cutoff value of negative control. The error bars represent the standard deviation of at least three independent repeated experiments for each assay.

The SNIA was found to work efficiently in detecting p24 in the linear working range of 0.02 pg/mL to 1000 pg/mL as observed in Fig. 5. This is one of the lowest concentration of HIV-1 p24 detected using an immunoassay. The dynamic range observed here is very useful for detecting p24 antigen concentrations within days after exposure upto 3 weeks, after which seroconversion renders the antigen undetectable^25^. Thus, this range is very significant in the early detection of the infection. A linear relation between the concentration of p24 and signal intensity was observed for the whole range tested. A very clear difference was observed between the measured signal intensity and the blank intensity until 0.02 pg/mL beyond which they could not be distinguished.

### Analytical performance of SNIA

The lower detection limit value was derived using the sum of the means of the signal intensity of six negative controls plus 2 times their standard deviation^33^ (SD) and found to be 20 fg/mL for SNIA. From the linear fit of the data a good correlation between the concentrations of p24 and the signal intensity by SNIA assay can be seen and the correlation coefficient was calculated to be R^2^ = 0.9954. This suggests SNIA is a linear dose dependent assay. In this paper, we report this fluorescent silica nanoparticle based immunoassay can achieve an analytical sensitivity of femtogram level, which is approximately thousand times higher in comparison with only picogram level sensitivity achieved with conventional colorimetric ELISA^17^.

### Precision and recovery rate of the assay

Our next objective was to study the applicability of SNIA in real world scenario by testing human plasma samples. The next step was to further evaluate the analytical sensitivity of the assay and this was done by taking known concentrations of p24 antigen and spiking the HIV negative plasma samples collected from healthy individuals (HIV negative sample). The plasma samples were diluted 100 times which in turn reduced the sample amount required for analysis. To the diluted plasma sample, purified p24 antigen concentrations of 0.5 pg/mL, 5 pg/mL, 50 pg/mL and 500 pg/mL were added. All experiments were conducted in triplicate and the concentration was calculated by taking the average signal response and comparing it with the standard curve (Fig. 5). It is clear from Table 2 that the quantification of the HIV-1 p24 antigen by SNIA yielded results that are comparable to the actual amounts spiked into plasma samples. Moreover, the table also shows that the present immunoassay could detect picogram levels of antigen with a sensitivity of 0.5 pg/mL in the the spiked plasma samples. This data suggest that this assay may be reliably adopted for the quantification of the HIV-1 p24 antigen.

**Table 2:** Recoveries of p24 antigen spiked into HIV negative plasma samples using SNIA.

Sample	Spiked concentration (pg/mL)	Measured Concentration (pg/mL)	Recovery (%)
1	0.5	0.53 ± 0.05	106.0 ± 10.0
2	5	4.79 ± 0.58	95.8 ± 11.6
3	50	48.17 ± 4.32	96.34 ± 8.64
4	500	506.0 ± 18.4	101.2 ± 3.6

### Clinical Serum Analysis

**Figure 6:**
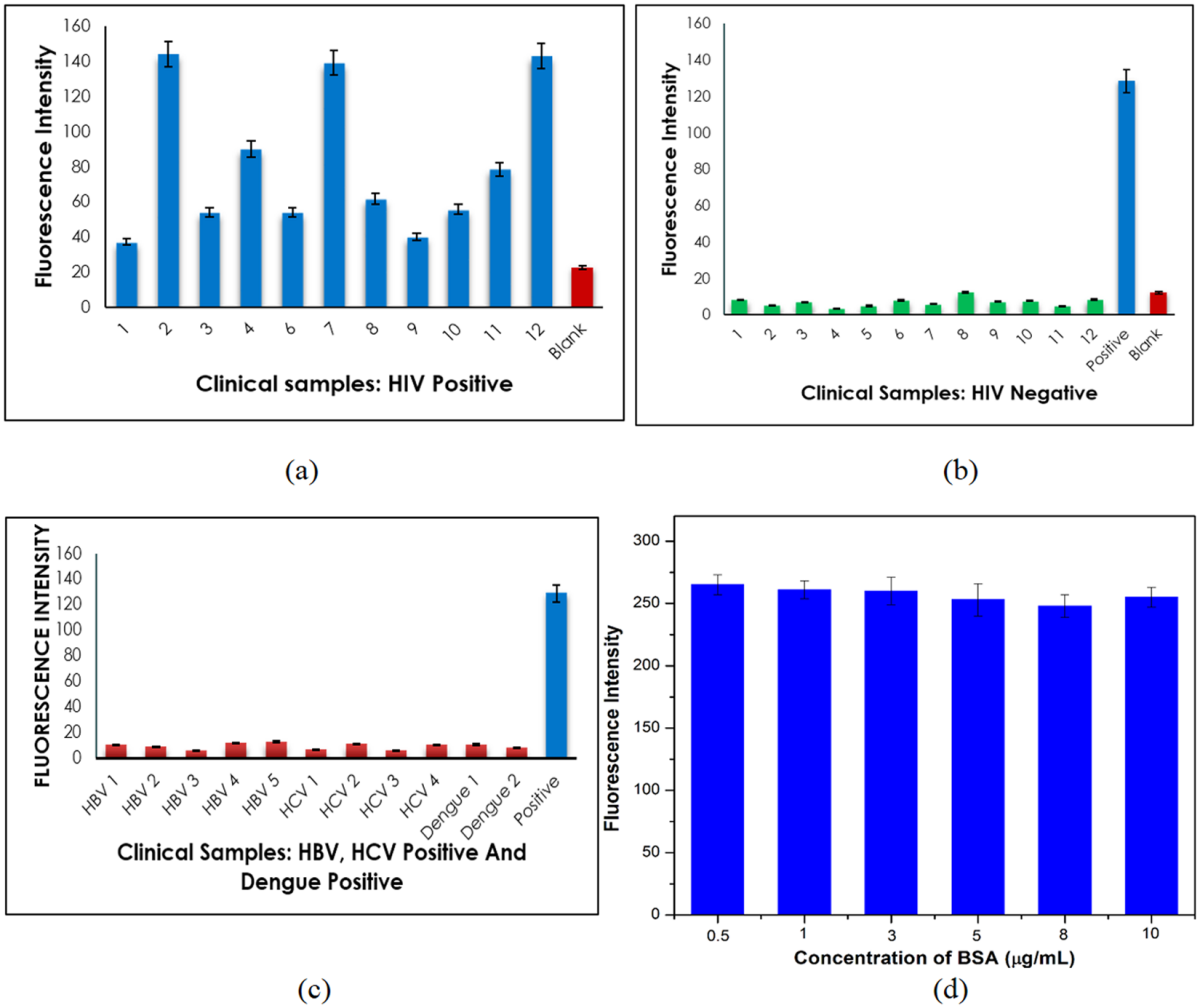
(**a**) SNIA applied to HIV positive samples and compared with the results from negative sample and blank. The blue and red bars denote the positive sample and blank. (**b**) SNIA applied to HIV negative samples and compared with the results from blank and HIV positive sample. The green, blue and red bars denote the negative, positive sample and blank respectively. (**c**) SNIA on HBV positive/HIV negative, HCV positive/HIV negative and dengue samples which are compared with the result from a HIV positive sample and a blank. The red bars denote the HBV positive/HIV negative, HCV positive/HIV negative, dengue samples and blue bars denote the lowest measured positive samples, respectively. (**d**) Evaluation of the effect of presence of BSA as an interfering protein on SNIA.

The specificity of the SNIA was assessed using plasma samples from HIV positive and negative individuals which was done by testing a total of 135 plasma samples. Samples from patients and healthy individuals were obtained from the Sri Sathya Sai Institute of Higher Medical Sciences, Prasanthigram, India, (Study Id-SSIHL/IEC/PSN/BS/2012/01). The details of the samples were coded and tested in a blinded manner. Samples were confirmed to be positive or negative using fourth generation Microlisa HIV Ag + Ab Elisa kit (J. Mitra & Co. Pvt. Ltd.). Out of the 135, 50 samples were taken from healthy individuals, which tested negative and no false positives were observed. Of the 55 confirmed HIV positive samples tested, no false negative results were observed (Fig. 6(a)). The mean ratio of samples over the cut-off ratio of the negative control for these samples was 107.8 ± 97.54. Further, we tested 50 plasma samples from confirmed HIV negative individuals which were confirmed by 3rd and 4th generation commercial ELISA kits. The signal to cut off ratio was considerably less than 14.00 (8.60 ± 3.62) and none of them were falsely positive Fig. 6(b). The specificity of the assay was further evaluated to test the interference of other viruses and cross reactivity in the detection of HIV-1 p24 by testing 10 HIV negative/HBV positive, 15 HIV negative/HCV positive and 5 HIV negative/Dengue positive plasma samples. The mean ratios of samples over the cut-off value of the negative control for samples from the patients HIV negative/HBV positive, HIV negative/HCV positive, HIV negative/Dengue positive infected samples were 8.96 ± 2.48, 10.47 ± 2.25, and 9.07 ± 2.1 respectively. The absence of false negative results in this study clearly indicates that there was no cross reactivity of HIV-1 p24 with HBV, HCV and Dengue viruses (Fig. 6(c)). Based on the above findings, the HIV-1 p24 SNIA exhibited a specificity of 100% (55/55). Also no crossreactivity was observed with HBV positive (10/10), HCV positive (15/15) and Dengue positive (5/5) samples.

### Effect of BSA as an interfering protein on SNIA

Another major problem which affects immunoassay is the interference of other biomolecules like plasma proteins, etc^34^. In order to test the stability of the immunoassay in the presence of interfering biomolecules, we performed SNIA in the presence of Bovine Serum Albumin (BSA). Six concentrations of BSA were taken in the range of 0.5 μg/mL–10 μg/mL, and its effect on the fluorescence intensity was observed at the p24 antigen concentration of 10 pg/mL. SNIA was performed in triplicate on these samples and the consequent effect of the interfering protein was studied. From Fig. 6(d) it is very clear that the fluorescent measurements are hardly affected by the presence of such an interfering protein.

Thus, we could confirm that the assay is very specific to p24 antigen and is not prone to cross reactivity effects from other biomolecules. As clearly evident from the Fig. 6, the SNIA has very high specificity and low cross reactivity.

These results indicate that the engineered streptavidin labelled Europium doped fluorescent silica nanoparticles based technology and their applications may provide a rapid and ultrasensitive testing platform for clinical diagnostics and laboratory research.

## Discussions

In this study we have demonstrated for the first time the feasibility of using fluorescent silica nanoparticles to detect HIV-1 p24 antigen at femtogram levels using an antigen based sandwich immunoassay (SNIA). These silica nanoparticles exhibited high photo stability, narrow emission widths and high quantum yield when compared to gold and dye doped nanoparticles. The characteristic large Stokes shift and time resolved fluorescence mode of detection by these nanoparticles were responsible in the reduction of background fluorescence which has enabled for the enhanced detection sensitivity. Apart from diagnostic applications, biocompatibility of silica nanoparticles is another important characteristic that may be useful for targeted drug delivery applications.

Present ultrasensitive SNIA may find applications in improved diagnosis and point-of-care use. The lower detection limit of HIV-1 p24 has been 15 pg/mL and 0.5 pg/mL for conventional ELISA and TSA-mediated signal amplification-boosted ELISA, respectively. A comaparison of various technologies (Table 3) for the detection of p24 antigen with the current SNIA reflect the high sensitivity of the present method. SNIA exhibited thousand fold analytical sensitivity when compared to traditional ELISA. The linear correlation between the target concentration and signal intensity observed can be used for quantitative detection of antigen in plasma samples. Because of high quantum yield of silica nanoparticles, this signal based amplification assay could achieve a sensitivity level close to PCR techniques which require sophisticated instruments, costly reagents and are very prone to contamination. SNIA turned out to be a very sensitive immunoassay because of its stable signal and high signal to noise ratio. As SNIA does not involve enzymatic reactions for detection, specific instrumentation for conducting reactions and special storage conditions for reagents are not required. Due to the high sensitivity of streptavidin conjugated silica nanoparticles, SNIA could be adopted and developed into a universal labelling technology for the detection of various pathogens and viruses by selecting appropriate capture and detection antibodies for the corresponding pathogen/virus. The methodology will remain the same which is an added advantage with the streptavidin labelling technology. Same set of conjugated nanoparticles can be used for testing for different antigens.Upon further optimization and simplification, SNIA could be developed into a rapid and ultrasensitive testing platform for clinical diagnosis and laboratory research in resource limited settings.

**Table 3:** Comparison of various techchnologies for the detection of p24 antigen.

S. No.	Method	P24 Concentration detected in pg/mL	Reference
1	Conventional Elisa	10–15	16
2	Inductively coupled plasma mass spectrometry based gold nanoparticle immunoassay	7.5	35
3	Europium nanoparticle based immunoassay	0.5	15
4	Capacitive Immunosensor	0.12	36
5	Microchip based Europium nanoparticle immunoassay	5	37
6	Single-molecule immunosorbent assay	0.1	38
7	ZnO nanoparticle based immunoassay	25	39
8	Fluorescent Silver nanoparticles immunoassay	10	40
9	P24 antigen detection assay with booster step	0.5	17
10	Immunofluorescent cytometric bead assay	0.4	41
11	Carbon Dots based immunoassay	20	42
12	Silica Nanoparticle based immunoassay	0.02	Current Study

## Conclusions

Our work has shown that the use of fluorescent silica nanoparticles in infectious disease assay development can result in highly enhanced assay sensitivity. Although there are similar assays, SNIA is capable of detecting HIV-1 p24 antigen in the femtogram range which will be useful in identifying more acute HIV infection cases. The findings described in this work will be important for those developing highly sensitive assays for the estimation and detection of acute HIV infection cases as part of the strategy towards an AIDS-free generation. This highly sensitive p24 assay can also help improve blood safety by reducing the antibody negative window period in blood donors in resource limited settings where nucleic acid testing is not practical or feasible.
